# PW01-023 – Ex vivo PBMC cytokine profile in FMF patients

**DOI:** 10.1186/1546-0096-11-S1-A76

**Published:** 2013-11-08

**Authors:** JN Ibrahim, R Jounblat, A Delwail, J Abou-Ghoch, N Salem, E Chouery, A Megarbane, J-C Lecron, M Medlej-Hashim

**Affiliations:** 1 Laboratoire inflammation, Tissus épithéliaux et cytokines; EA4331, University of Poitiers, Poitiers, France; 2 ER030-EDST; Faculty of Sciences II, Lebanese University, Beirut, Lebanon; 3 Medical Genetics Unit, Faculty of Medicine, Saint Joseph University, Beirut, Lebanon

## Introduction

Familial Mediterranean fever (FMF) is an autosomal recessive autoinflammatory disorder affecting mainly Jews, Armenians, Turks and Arabs. Recently, several pro- and anti-inflammatory cytokines have been studied in sera of Armenian and Turkish FMF patients during and in between crises. However, the information were limited and contradictory.

## Objectives

This work aimed to evaluate the *ex vivo* and serum cytokine profile of Lebanese FMF patients during acute attacks and attack-free periods and to compare it with that of healthy controls in order to identify a specific cytokine ¨signature¨ and to understand the role of the inflammasome in this autoinflammatory disease.

## Methods

The study included 34 FMF patients, of whom 9 were studied during both attack and remission and 25 healthy controls. Cytokine levels were evaluated in serum and supernatants of peripheral PBMC cultures with and without 24h stimulation of monocytes by LPS and T lymphocytes by anti-CD3/CD28 beads, by Luminex Multiplex ELISA.

## Results

### Ex vivo cytokine profile

The levels of pro-inflammatory cytokines IL-6 and TNF-α were higher in unstimulated and LPS stimulated PBMC supernatants of FMF patients in crises compared to the control group. Concentrations were comparable between FMF patients during and between crises.

There was no difference in spontaneous IL1-β and IL-1 α release by PBMCs of FMF patients and controls. However, in response to LPS stimulation, levels of these cytokines were found higher in PBMC supernatants of FMF patients during crises compared to those in remission and to the controls. In contrast, no difference was found in IL1-RA levels between FMF patients and controls in all conditions.

Regarding Th1 and Th2 cytokines, IFN-γ and IL-4 levels were lower in unstimulated and anti-CD3/CD28 stimulated PBMCs supernatants of FMF patients during and between crises compared to the controls. Moreover, lower levels of those cytokines were detected in culture supernatants of FMF patients during crises compared to those in remission after T cell stimulation.

For Th17 cytokines, IL-17 was higher in anti-CD3/CD28 stimulated PBMC supernatants of FMF patients during crises compared to the control group. After T cell stimulation, PBMCs from FMF patients in remission release more IL-22 than PBMCs from control subjects.

Finally, no difference in IL-10 levels was detected between FMF patients and controls.

### Serum cytokine profile

Except IL-6, all cytokines tested, were almost not detected in the serum of patients and controls.

## Conclusion

The cytokine changes observed in FMF patients and characterized by a continuous pro-inflammatory Th17 and IL-1 family cytokine activation and a reduced Th1 and Th2 response, suggest an ongoing subclinical inflammation and represent a specific cytokine ¨signature¨ to FMF patients. In addition, these results do not show a particular involvement of the inflammasome in FMF physiopathology. Finally, we suggest that *ex vivo* study represents a novel and interesting approach to evaluate the cytokine involvement in FMF patients.

## Disclosure of interest

None declared

